# Detection of Zoonotic *Bartonella* Pathogens in Rabbit Fleas, Colorado, USA

**DOI:** 10.3201/eid2604.191161

**Published:** 2020-04

**Authors:** Shingo Sato, R. Jory Brinkerhoff, Erin Hollis, Shunta Funada, Avery B. Shannon, Soichi Maruyama

**Affiliations:** Nihon University College of Bioresource Sciences, Fujisawa, Japan (S. Sato, S. Funada, S. Maruyama);; University of Richmond, Richmond, Virginia, USA (R.J. Brinkerhoff, E. Hollis, A.B. Shannon);; University of KwaZulu-Natal, Pietermaritzburg, South Africa (R.J. Brinkerhoff)

**Keywords:** disease ecology, lagomorpha, Siphonaptera, Bartonella, rabbit fleas, vector-borne infections, zoonoses, Colorado, bacteria, United States

## Abstract

We detected 3 *Bartonella* species in wild rabbit fleas from Colorado, USA: *B. vinsonii* subsp. *berkhoffii* (n = 16), *B. alsatica* (n = 5), and *B. rochalimae* (n = 1). Our results support the establishment of the zoonotic agent *B. alsatica* in North America.

Wild lagomorphs (rabbits, hares, and pikas) are known or potential reservoirs for a number of zoonotic agents, including tularemia (*Francisella tularensis*), plague (*Yersinia pestis*), pasteurellosis (*Pasteurella multocida*), ringworm (*Trichophyton* spp.), and cryptosporidiosis (*Cryptosporidium cuniculus*) (*1*). In 1999, a novel *Bartonella* species, *B. alsatica*, was isolated from the blood of wild rabbits (*Oryctolagus cuniculus*) in eastern France (*2*). *B. alsatica* was later identified as a causative agent of lymphadenitis (*3*) and endocarditis (*4*,*5*) in humans. A case of prosthetic vascular graft infection caused by *B. alsatica* was reported in 2019 (*6*). 

The geographic distribution of *B. alsatica* is poorly understood, as is its mode of transmission, although vector-mediated transmission was suggested upon initial characterization of this agent (*2*). *B. alsatica* DNA has been detected in fleas collected from *Bartonella*-infected wild rabbits in France (*7*) and Spain (*8*,*9*), suggesting the potential for fleaborne *B. alsatica* transmission. Our goal was to describe associations between rabbit-associated *Bartonella* and potential flea vectors in the United States to gain insights into transmission of fleaborne zoonoses.

## The Study

We collected fleas from live-trapped desert cottontail rabbits (*Sylvilagus audubonii*) in June and July 2005 from 8 sites in Boulder County, Colorado, USA (*10*). We identified fleas to species by light microscopy using dichotomous keys (*10*) and then stored them in 96-well tissue culture plates at −20°C, except for representatives of each species that were removed and submitted to the Centers for Disease Control and Prevention (Fort Collins, CO, USA). In 2015, we extracted DNA from individual fleas using commercial DNA extraction kits (Blood and Tissue Kit; Macherey-Nagel, Inc., https://www.mn-net.com), with aliquots of extracted DNA maintained at the University of Richmond (Richmond, VA, USA), and secondary aliquots sent to the Laboratory of Veterinary Public Health, Nihon University College of Bioresource Sciences (Fujisawa, Japan). Both laboratories screened samples for *Bartonella* infection by conventional PCR targeting part of the *ssrA* gene; primers used were ssrA-F (5′-GCTATGGTAATAAATGGACAATGAAATAA-3′) and ssrA-R (5′-GCTTCTGTTGCCAGGTG-3′). The targeted gene was selected because of the robustness of the PCR assay and the ability of the locus to segregate *Bartonella* at the species level (*11*). Nihon University College of Bioresource Sciences also used real-time PCR targeting the *ssrA* gene to confirm *Bartonella* species for those samples. This PCR used a genus-specific TaqMan probe, 6-carboxyfluorescein (FAM)–labeled probe (5′-FAM-ACCCCGCTTAAACCTGCGACG-3′-BHQ1, where BHQ is black hole quencher); primers were the same as for conventional PCR. Samples that tested positive for *Bartonella* DNA by real-time PCR and for which unambiguous sequence data were collected in both laboratories from the target locus (*ssrA*) were reported as *Bartonella* positive. We sequenced all amplicons (301 bp) from conventional PCR and aligned them with *Bartonella* type strains and then subjected them to phylogenetic analysis using MEGA 7.0 (https://www.megasoftware.net).

We collected 141 fleas from 14 desert cottontail rabbits (average fleas per parasitized host 14.3, range 1–54) in the summer of 2005. Of these fleas, 105 (81 *Euhoplopsyllus glacialis* and 24 *Cediopsylla inaequalis*) collected from 7 rabbits sampled at 4 sites (Table 1; specific site locations in *10*) were available for molecular screening for *Bartonella.* The remaining 36 fleas were processed for *Yesinia pestis* surveillance in a separate project (R.J. Brinkerhoff et al., unpub. data) and were not available for *Bartonella* testing. 

**Table 1:** Fleas collected from desert cottontail rabbits (*Sylvilagus audubonii*) in Boulder County, Colorado, USA, and analyzed for presence of *Bartonella*

Rabbit ID no.	Sampling date	No. fleas collected (no. tested)	No. *Bartonella*-positive fleas (no. tested), % positive		*Bartonella* prevalence in fleas, %
			*Cediopsylla inaequalis*	*Euhoplopsyllus glacialis*	
304	2005 Jul 14	51 (21)	0 (1), 0	0 (20), 0	0
305	2005 Jul 14	2 (2)	0	1 (2), 50	50.0
522	2005 Jul 18	55 (53)	2 (19), 11	6 (34), 18	15.1
633	2005 Jul 18	17 (17)	0 (4), 0	11 (13), 85	64.7
673	2005 Jul 21	4 (2)	0, 0	1 (2), 50	50.0
674	2005 Jul 21	8 (7)	0, 0	2 (7), 29	28.6
794	2005 Jul 28	4 (3)	0, 0	0 (3), 0	0
Total		141* (105)	2 (24)	21 (81)	21.9

*Thirty-six fleas had been processed for *Yesinia pestis* surveillance in a previous project (R.J. Brinkerhoff et al., unpub. data) and thus were not tested for this study.

We detected *Bartonella* DNA in 2 (8.3%) *C. inaequalis* fleas collected from 1 rabbit (ID no. 522) and 21 (25.9%) *E. glacialis* fleas collected from 5 rabbits (ID nos. 305, 522, 633, 673, and 674) (Table 1). All nucleotide sequences matched closely to 3 zoonotic *Bartonella* species, *B. alsatica*, *B. vinsonii* subsp. *berkhoffii*, and *B. rochalimae* (Table 2), and clustered phylogenetically with reference sequences of the type strains with high bootstrap support (Figure). The representative sequences of the 3 *Bartonella* species were registered in International Nucleotide Sequence Database Collaboration with accession nos. PS522-c9 (GenBank accession no. MN654366), PS674-e5 (GenBank accession no. MN654366), and PS674-e6 (GenBank accession no. MN654366). All 3 rabbits (ID nos. 522, 633, 674) from which >1 flea was PCR-positive and available for sequencing produced multiple *Bartonella* species (Table 2).

**Table 2:** *Bartonella* sequence identities for *ssrA* amplicons amplified from *Cediopsylla inaequalis* and *Euhoplopsyllus glacialis* fleas collected from 5 desert cottontail rabbits (*Sylvilagus audubonii*) in Boulder County, Colorado, USA*

Host no.	Flea nos.	Flea species	Closest *Bartonella* strains/sequence homologies/% (GenBank accession no.)
305	e1	*E. glacialis*	*B. vinsonii* subsp. *berkhoffii* strain Winnie/98.8% (CP003124)
522	e6, e25, e26, e29	*E. glacialis*	*B. v.* subsp. *berkhoffii* strain Winnie/98.8% (CP003124)
	e12	*E. glacialis*	*B. alsatica* strain IBS 382/96.9% (JN029776)
	e32	*E. glacialis*	*B. alsatica* strain IBS 382/96.9% (JN029776)
	c1	*C. inaequalis*	*B. v.* subsp. *berkhoffii* strain Winnie/98.8% (CP003124)
	c9	*C. inaequalis*	*B. rochalimae* strain BMGH (JN029797) 100%
633	e1, e2, e3, e4, e5, e8, e9, e10, e11	*E. glacialis*	*B. v.* subsp. *berkhoffii* strain Winnie/98.8% (CP003124)
	e12	*E. glacialis*	*B. alsatica* strain IBS 382/96.9% (JN029776)
	e13	*E. glacialis*	*B. alsatica* strain IBS 382/96.9% (JN029776)
673	e1	*E. glacialis*	*B. alsatica* strain IBS 382/96.9% (JN029776)
674	e5	*E. glacialis*	*B. v.* subsp. *berkhoffii* strain Winnie/98.8% (CP003124)
	e6	*E. glacialis*	*B. alsatica* strain IBS 382/96.9% (JN029776)

*All samples in the table tested positive for *Bartonella* DNA by *ssrA* real-time PCR (*11*), whereas no samples that were negative by conventional *ssrA* PCR tested positive by real-time PCR.

**Figure:**
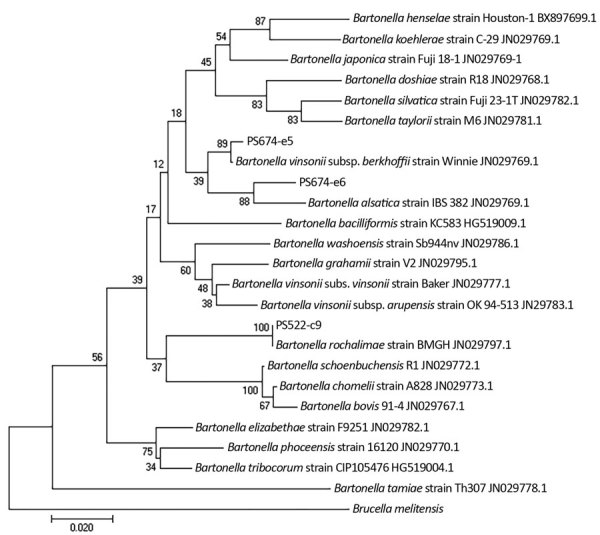
Phylogenetic relationships of *Bartonella ssrA* sequences detected in study of zoonotic *Bartonella *in rabbit fleas, Colorado, USA, compared with reference sequences. This tree was generated based on 253 bp by maximum likelihood and 1,000 bootstrap replicates using the Kimura 2-parameter evolutionary model with gamma-distributed rates among sites. Sample numbers are found in Table 2. GenBank accession numbers are indicated. Scale bar indicates nucleotide substitutions per site.

## Conclusions

We detected DNA of 3 zoonotic *Bartonella* species among the 105 rabbit fleas we tested for this study; overall *Bartonella* prevalence in fleas was 21.9% (23/105), which is comparable to previous prevalence estimates from rabbit fleas in Europe (*7*,*9*). This study had several noteworthy findings: *B. alsatica* DNA was detected in North America, and carnivore-associated *Bartonella* species occurred in rabbit fleas. These findings highlight the complexity of pathogen–vector–host associations and demonstrate why vector ecology is necessary for elucidating the evolution and enzootic transmission of vectorborne pathogens. Since *B. alsatica* was described in 1999 (*2*), its DNA has been detected not only in European rabbits (*Oryctolagus cuniculus*) in Spain (*8*) but also in rabbit fleas (*Spilopsyllus cuniculi* and *Xenopsylla cunicularis*) collected in France (*7*) and Spain (*9*) and has been associated with human disease in France (*3*–*6*). In 2019, detection of *B. alsatica* DNA was reported in cat fleas (*Ctenocephalides felis*) infesting cats and dogs in the United Kingdom (*12*). 

A recent study reported *ftsZ* and *nuoG* sequences with ≈95% similarity to *B. alsatica* from the spleens of spiny rats (*Thrichomys fosteri*) in Brazil (*13*), the only previous published report of *B. alsatica* in the Americas. The *B. alsatica* sequences in our study were more similar to the *B. alsatica* type strain than were the putative *B. alsatica* sequences detected in Brazil. However, the *B. alsatica* sequences in our study were not identical to the type strain, suggesting that divergent *B. alsatica* strains may be circulating in the Americas. Further sampling of lagomorphs and their ectoparasites throughout North and South America is necessary to determine the geographic extent of *B. alsatica*, as well as its genotypic variation and evolutionary history.

We can conclude that both *C. inaequalis* and *E. glacialis* fleas are able to acquire *Bartonella* DNA and that blood-feeding is a likely mode of *Bartonella* acquisition, based on the observation that multiple fleas from the same host tested positive for *Bartonella* DNA. The detection of carnivore-associated *Bartonella* species in rabbit fleas was unexpected; typical reservoirs for *B. rochalimae* and *B. v.* subsp. *berkhoffii* are wild carnivores such as coyotes, foxes, raccoons, and skunks. However, *B. rochalimae* or *B. rochalimae*–like bacteria were found in the blood of brown rats (*Rattus norvegicus*) captured in Taiwan and in California, USA (*14*). Thus, *B. rochalimae* might have the potential to infect rodents as well as carnivores. Both rabbit flea species sampled in this study have been recovered from wild carnivore species in our study system and thus could serve as bridge vectors between carnivores and rabbits (*10*). In Europe, rabbit fleas have also been collected from carnivores (*15*), suggesting potential lagomorph–carnivore *B. alsatica* transmission in other systems as well.

*Yersinia pestis*, another fleaborne zoonotic agent that periodically causes epizootic events in our system (*10*), may spill over into amplifying hosts from putative reservoirs (mammalian, flea, or both) or from environmental sources. Flea and *Bartonella* (Table 2) exchange between lagomorphs and carnivores suggests that *Y. pestis* could also jump among these groups of mammals. Moreover, desert cottontails co-occur with black-tailed prairie dogs (*Cynomys ludovicianus*), a species associated with epizootic *Y. pestis* emergence, and flea exchange between desert cottontails and prairie dogs has been described (*10*). Given our findings, it is apparent that desert cottontail rabbits are associated with multiple zoonotic *Bartonella* species, including *B. alsatica*, which had not been previously recorded in North America, and that wild lagomorphs may contribute to the maintenance and transmission of several vectorborne zoonoses.
